# Stentless Freestyle Root Replacement: Distillation of Technique From a 30-Year Experience

**DOI:** 10.1016/j.atssr.2023.11.002

**Published:** 2023-11-21

**Authors:** Bartlomiej R. Imielski, Gabriel E. Cambronero, Zohaib R. Khawaja, Yusuf M. Aboutabl, Adrian L. Lata, Edward H. Kincaid, Neal D. Kon

**Affiliations:** 1 Department of Cardiothoracic Surgery, Wake Forest School of Medicine, Winston-Salem, North Carolina

## Abstract

In 1968, Bentall introduced a composite valve-graft that became the “gold standard” for aortic root replacement. Since then, several alternatives have arisen; one is the Freestyle (Medtronic) stentless aortic root bioprosthesis. This porcine aortic valve has excellent results, and we describe the surgical technique we have developed during our 30-year experience.

Bentall advanced aortic surgery with a composite valve-graft, ushering in the “gold standard” for aortic root replacement.^1^ Root surgery has progressed to include valve-sparing reconstruction, homografts, pulmonary autografts, and stented bioprostheses. These face challenges, such as availability, technical demand, and impaired hemodynamics secondary to a stented graft in a smaller human root.^2^^,^^3^ We commonly use the Freestyle stentless aortic root bioprosthesis (Medtronic). Approved for human implantation in 1992, the Freestyle graft has excellent hemodynamics and long-term outcomes.^3^^,^^4^

## Technique

### Setup

After median sternotomy, aortic cannulation is performed just below the innominate artery. Venous cannulation is performed through the right atrial appendage with a dual-stage cannula. A left ventricular vent is placed in the right superior pulmonary vein. Cardiopulmonary arrest is initiated. Despite the presence of a vent, the left ventricle is monitored for distention due to aortic regurgitation, which may necessitate early aortic transection and direct ostial cardioplegic arrest. We systemically cool patients to 32 °C.

### Preparation of the Root

The aorta is transected along the greater curve, continued in a longitudinal aortotomy toward the cross-clamp, stopping 1 cm short. Dissection is carried out circumferentially, with care taken not to cut toward the inferior aspect of the cross-clamp. The clamped portion of the aorta is freed from pericardial attachments and off the right main pulmonary artery. The proximal ascending aorta is resected to the sinotubular junction. The root is inspected. At this juncture, the surgeon can still choose between an aortic valve replacement, aortic root replacement, and valve-sparing root reconstruction. Once commitment to a root replacement is made, the valve leaflets are excised and the annulus is débrided.

Dissection of the coronary buttons follows. The root is mobilized from its circumferential epicardial attachments. Once it is mobilized to the level of the coronaries, the right coronary button (RCB) is dissected. The root is incised halfway between the commissures and the button in a straight downward trajectory, ensuring that sufficient tissue remains on both sides for suturing. After cutting down on both sides of the button, a U cut is made and the bottom freed from its most inferior attachment. The RCB is further mobilized from the root and suspended by a pledgeted 5-0 Prolene suture. Similarly, the left coronary button (LCB) is dissected free from the root. Additional freedom is achieved by releasing the LCB from its pericardial attachment to the right main pulmonary artery.

The formal root dissection continues after coronary button dissection. The root is dissected to its base, including division of the conus ligament to the pulmonary valve apparatus, down to the myocardium of the right ventricular outflow track, and off the dome of the left atrium. At the nadir of this dissection, small veins below the coronary buttons may be encountered that may bleed when tested with cardioplegia down the ostia; therefore, they should be suture ligated with 7-0 Prolene sutures. Any inadvertent injury to the dome of the left atrium or right atrial appendage should be fixed now. The xenograft has larger native sinuses than human sinuses, which must be considered during root dissection.

Annular preparation continues by resecting the noncoronary sinus, leaving approximately 5 to 7 mm of aortic tissue. The 3 commissures are suspended to maximize root visualization.

### Sizing (“One Size Up”)

With so much emphasis on patient-prosthesis mismatch, outflow gradients, and perivalvular leaks, proper and reproducible sizing is paramount. The Freestyle sizers correspond to the left ventricular outflow tract internal diameter. We upsize by 2 mm or 1 graft size larger than the sizer that falls into the left ventricular outflow tract. Undersizing a Freestyle graft increases suture line tension and impairs hemodynamic performance. Oversizing will cause infolding and crimping of the valve apparatus because of its stentless nature, leading to worsened gradients, impaired performance, and reduced durability.

Next, nonpledgeted valve sutures are used with a suture stadium in a single interrupted circumferential fashion. The proper suture spacing mimics the number of skirt sutures/dots on the graft. For example, a 23-mm Freestyle has 29 skirt sutures/dots, which perfectly correlates to the number of valve annular sutures needed—29. We divide the annulus into thirds and begin with 3 commissural valve sutures. The remaining sutures are spaced into thirds. We caution against mattress sutures, especially pledgeted ones, as they narrow the inflow suture line. The use of the Cor-Knot automated fastener (LSI Solutions) has markedly facilitated implantation.

### Freestyle Inspection

The most frequent question is how to align the xenograft sinuses. Understanding the differences between the porcine and human root helps. The largest porcine sinuses of Valsalva are the left and right; for humans, the largest is the noncoronary sinus. The human root has the left and right sinuses of Valsalva 90° to 110° apart, whereas the porcine sinuses of Valsalva are 120° to 140° apart. Therefore, for a trileaflet valve, the simplest solution to orienting the xenograft is to rotate the graft 120°.^5^ As such, the porcine noncoronary, left, and right sinuses correspond to the human left, right, and noncoronary sinuses, respectively. The final step in root realignment is to clip the oversewn porcine right coronary artery remnant as a precaution. If the human left and right sinuses of Valsalva are farther apart, as with bicuspid valves, the xenograft does not require rotation and is implanted in its morphologic orientation.

On orientation, the ventricular sutures are passed through the xenograft in an in-to-out fashion. Once completed circumferentially, the xenograft is slid down toward the root, seated to ensure adequate apposition, and secured using the Cor-Knot fastener. After all sutures are secured, the anastomotic suture line is inspected as now is the time to fix issues, before obstruction from reimplanted coronary buttons.

### Coronary Button Reimplantation (Left, Graft, Right)

Proceeding with the LCB, the location is measured and the neo-ostium is sharply created. A running suture is started with 5-0 Prolene. The inferior aspect of the button is run while ensuring that the needle stays outside the coronary because a tendency exists to suture inside the bend of the ostium. The anastomosis is completed circumferentially. Emphasis is placed on rolling the needle through this delicate tissue, not pushing or pulling. If there is ample time, the anterior aspect of the anastomosis is reinforced with interrupted fix-it stitches or a second running 5-0 Prolene suture.

Next, attention is turned to the Freestyle–distal aortic suture line. Completing this anastomosis next ensures proper root alignment for the RCB, ensuring it will not kink. The graft-to-aortic suture line is completed with 5-0 or 4-0 Prolene, depending on tissue quality. Well-spaced, full-thickness bites that incorporate the adventitia are stressed. If the graft does not adequately reach the aorta, an interposition graft can be used.

Reimplantation of the RCB is completed in the same fashion as for the LCB; however, 2 pearls exist. If additional length is required, the first branch of the right coronary artery, the conus or sinoatrial, may be divided as a last resort. Conversely, if there is a large root aneurysm and a smaller graft, careful attention is paid to the RCB orientation to prevent kinking or twisting.

The remainder of the operation follows standard practices, including deairing, weaning off of cardiopulmonary bypass, and pacing.

## Comment

In our experience, this technique results in durable, hemodynamically well-functioning grafts that offer great versatility regardless of indication—aneurysm, dissection, or endocarditis. This xenograft mitigates the need for early anticoagulation, given its lack of a sewing cuff. The pledget-free implantation further minimizes gradients and prevents pannus formation. Last, we prefer the 27-mm to the 29-mm Freestyle, as our experience demonstrates that the 27-mm graft has better results if a subsequent valve-in-valve transcatheter aortic valve replacement is required, which has excellent procedural success and outcomes.^6^
